# Can current national surveillance systems in England and Wales monitor sexual transmission of hepatitis C among HIV-infected men who have sex with men?

**DOI:** 10.1186/1471-2458-7-7

**Published:** 2007-01-18

**Authors:** Sarah Dougan, M Adekoyejo Balogun, Jonathan Elford, Lisa J Brant, Katy Sinka, Barry G Evans, Mary E Ramsay

**Affiliations:** 1 HIV and STI Department, Health Protection Agency Centre for Infections, London, UK; 2 City University London, Institute of Health Sciences, St Bartholomew School of Nursing and Midwifery, UK; 3 Immunisation Department, Health Protection Agency Centre for Infections, London, UK

## Abstract

**Background:**

Recent reports suggest an increase in sexually-transmitted hepatitis C infection among HIV-infected men who have sex with men (MSM) in European cities. We investigated whether current national surveillance systems in England and Wales (E&W) are able to monitor sexual transmission of hepatitis C infection among HIV-infected MSM.

**Methods:**

Routine laboratory reports of hepatitis C diagnoses and data from sentinel hepatitis C testing surveillance were matched to HIV diagnosis reports to determine: (i) the number of MSM diagnosed with HIV *and *hepatitis C (1996–2003); (ii) the number of HIV-diagnosed MSM tested for hepatitis C and found to be positive at sentinel sites (2003).

**Results:**

(i) Between 1996–2003, 38,027 hepatitis C diagnoses were reported; 25,938 (68%) were eligible for matching with HIV diagnoses. Thirty-one men (four in London) had both a HIV and hepatitis C diagnosis where the *only *risk was sex with another man. Numbers of "co-diagnosed" MSM increased from 0 in 1996 to 14 in 2003. The majority of MSM (22/31) tested hepatitis C positive after HIV diagnosis. (ii) Of 78,058 test results from sentinel hepatitis C testing sites in 2003, 67,712 (87%) were eligible for matching with HIV diagnoses. We identified 242 HIV-diagnosed MSM who did not inject drugs who tested for hepatitis C in 2003; 11 (4.5%) tested hepatitis C positive (95%CI: 2.3%–8.0%). Applying this percentage to all MSM seen for HIV-related care in E&W in 2003, an estimated 680 MSM living with diagnosed HIV would have tested positive for sexually-transmitted hepatitis C (95%CI: 346–1208).

**Conclusion:**

Matching routine laboratory reports of hepatitis C diagnoses with HIV diagnoses only identified 31 HIV infected MSM with sexually-transmitted hepatitis C infection. Clinical studies suggest that this is an underestimate. On the other hand, matching sentinel surveillance reports with HIV diagnoses revealed that in E&W in 2003 nearly 5% of HIV-diagnosed MSM tested hepatitis C positive where the only risk was sex with another man. Reports of sexually-transmitted hepatitis C infection were not confined to London. Enhanced surveillance is needed to monitor sexually-transmitted hepatitis C among HIV-infected MSM in E&W.

## Background

There have been recent reports of an increase in sexually-transmitted hepatitis C infection among HIV positive men who have sex with men (MSM) in London and other European cities [1-5]. Historically, sex between men has accounted for relatively few cases of hepatitis C, with most hepatitis C infections being acquired through injecting drug use [6]. National surveillance of HIV and hepatitis C infections reflects this historical picture, with diagnoses of HIV and hepatitis C and sentinel surveillance of hepatitis C testing being monitored by separate systems. For reasons of confidentiality, HIV status is not recorded for hepatitis C diagnoses, and hepatitis C status has not been systematically collected for HIV diagnoses. Co-infection is only routinely monitored for injecting drug users through an unlinked anonymous survey [7].

Evidence of an increase in sexually transmitted hepatitis C infections among HIV-infected MSM in England and Wales (E&W) comes from clinical studies only, mainly confined to London and the south east of England [1,8]. At a central London sexual health clinic, Browne *et al *identified 26 HIV positive MSM with sexually transmitted hepatitis C infection between 1997 and 2002. More recently, between October 2002 and August 2005, Danta *et al *reported on 225 HIV positive MSM with sexually transmitted hepatitis C seen in six large London genito-urinary medicine (GUM) clinics and one in Brighton [1,8]. Hepatitis C infections among HIV positive MSM appear to be associated with the following: unprotected anal intercourse; non-injecting drug use; concurrent sexually transmitted infections; and mucosally traumatic practices such as fisting, which may result in parenteral transmission [2,4,5,8]. HIV infection itself, may facilitate hepatitis C infection by promoting viral receptivity and increasing levels of hepatitis C RNA in semen [9,10]. Transmission of hepatitis C among HIV-negative MSM however, still appears to be rare [11,12].

While clinical studies suggest a rising number of sexually transmitted hepatitis C infections among previously diagnosed HIV-infected MSM it is unclear as to whether this is a national phenomenon and, if so, the extent to which this is happening across E&W. Unfortunately there is currently no single national surveillance system that can monitor HIV-hepatitis C co-infection among MSM. Since the same patient information (soundex code of surname [13], date of birth (DOB) and sex) is collected on reports however, there may be an opportunity to identify individuals who appear in both HIV and hepatitis C surveillance datasets by 'matching' individual reports.

In this exploratory study using national surveillance data, we investigate whether existing national HIV and hepatitis C surveillance systems can be used to estimate the number of HIV-infected MSM in E&W diagnosed with sexually transmitted hepatitis C in two ways. First, we try to match individual case reports of HIV and hepatitis C diagnoses between 1996–2003. Secondly, we try to match case reports of HIV diagnoses with *all *hepatitis C test results from laboratories participating in a sentinel surveillance study during 2002–2003. This is the first time these matching exercises have been conducted in E&W to examine sexually transmitted hepatitis C among HIV-infected MSM.

## Methods

### Data sources

#### HIV diagnoses

Reports of HIV diagnoses are received by the Health Protection Agency (HPA) Centre for Infections from laboratories (since 1985) and clinics (since 2000); the latter also report new AIDS diagnoses (since 1982). Patient information (soundex code of surname [13], DOB, sex) is collected on all reports, enabling the identification of multiple reports of the same individual without revealing their identity or compromising confidentiality. A report cannot be entered onto the system until all patient information is complete. Missing information is followed up with the laboratory or clinic. Probable route of infection is also collected on all reports (i.e. sex between men, sex between men and women, injecting drug use, blood transfusion etc), and followed up where incomplete.

#### Hepatitis C diagnoses

Laboratory confirmed cases of hepatitis C have been routinely reported to the HPA Centre for Infections since the early 1990s. Patient information (soundex code [13], DOB, sex) is collected along with the likely route of infection, reporting laboratory and region of diagnosis. Unlike HIV diagnoses, laboratory-confirmed hepatitis C cases are *not *routinely followed up where information is missing, so some reports do not have complete information on soundex code, DOB, sex and how the infection was acquired. A laboratory case is confirmed by the detection of antibody to HCV (anti-HCV) or HCV RNA in serum. Current available laboratory assays for HCV infection cannot distinguish between acute and chronic infections. While requests have been made for information on symptomatic acute infections to be provided on laboratory reports since 1996, this is rarely available.

#### Sentinel surveillance of hepatitis C testing

In the sentinel surveillance study of hepatitis C testing, data were collected on all hepatitis C tests (negative, positive, equivocal) undertaken in eight laboratories (two in London, six elsewhere in England) between January 2002 and December 2003 only [14]. Patient information (soundex code, DOB, sex) was collected for each individual but was not followed up where missing. As for the routine surveillance of hepatitis C diagnoses, a case was confirmed by the detection of antibody to HCV (anti-HCV) or HCV RNA in serum.

### Matching exercise

#### (i) Matching individual HIV and hepatitis C diagnoses  

Laboratory reports of hepatitis C diagnoses made between January 1996 and December 2003 (reports received by the end of May 2004) were matched to reports of HIV diagnoses since reporting began in 1982 through to December 2003 (reports received by end of September 2004). Reports without enough patient information were excluded. Exact matching was undertaken, for example, where reports with exactly the same soundex code, DOB, sex and reporting laboratory were identified. Further matching was also undertaken to allow for errors in data transcription. For example, soundex code, sex, reporting laboratory, month and year of birth would be matched exactly, allowing for errors in the transcription of the day of birth. All matches were verified by eye.

#### (ii) Matching individual HIV diagnoses to reports from sentinel hepatitis C testing  

Reports from the sentinel surveillance of hepatitis C testing from January 2002 to December 2003 were also matched to reports of HIV diagnoses using the procedure described above.

MSM identified with both hepatitis C and HIV diagnoses are described as "co-diagnosed". Probable route of infection is recorded in both the HIV and hepatitis C laboratory diagnoses datasets and in the sentinel surveillance of hepatitis C testing. Co-diagnosed MSM were assumed to have acquired their hepatitis C infection as a result of sex with another man if no other risks (e.g. injecting drug use) were reported in either dataset. Men with other risks were excluded from the analysis.

### Patient confidentiality and ethics

In England and Wales, reports of HIV diagnoses and diagnoses of hepatitis C infection are voluntary and confidential. To maintain patient confidentiality no names are held on the HIV database; soundex codes (a pseudonomyised code of a surname) are used instead [13]. The reporting systems have approval under the section 60 regulations of the Health and Social Care Act 2001 (Statutory Instrument 1438 – June 2002). All data are stored on restricted and secure databases at the HPA, with strict adherence to the Data Protection Act and Caldicott Guidelines [15]. Ethical approval was obtained for the sentinel surveillance of hepatitis C testing from the Northern and Yorkshire Multi-Centre Research Ethics Committee (MREC1/3/76) and the Public Health Laboratory Service Ethics Committee.

## Results

### Matching individual HIV and hepatitis C diagnoses

Of the 38,027 hepatitis C infections diagnosed between 1996 and 2003 and reported to the HPA, 68% (25,938/38,027) were eligible for inclusion in the matching exercise. The number and proportion of hepatitis C laboratory reports eligible for inclusion rose over time, from 50% (1,256/2,499) in 1996 to 74% (4,749/6,448) in 2003. The number of reports and degree of matching varied by region (table 1).

**Table 1:** Number of HIV diagnoses, hepatitis C laboratory diagnoses and reports from the sentinel surveillance of hepatitis C testing received and eligible for inclusion in the matching exercise by region of diagnosis/test

**Region of diagnosis/test**	**HIV diagnoses (1982–2003)**	**Hepatitis C laboratory diagnoses (1996–2003)**			**Sentinel surveillance of hepatitis C testing (2002–2003)**		
	
	No of diagnoses	No of reports	No included in the matching exercise*		No of reports	No included in the matching exercise*	
					
			n	*%*		n	*%*
East Midlands	1675	1721	1017	*59*	8337	7927	*95*
Eastern	2561	3859	2488	*64*	na	na	*na*
London	35401	2630	1822	*69*	3187	2959	*92*
North East	848	926	693	*75*	11814	9848	*83*
North West	3840	8107	6280	*77*	31576	27381	*87*
South East	5514	5655	3615	*64*	na	na	*na*
South West	1937	5748	4441	*77*	na	na	*na*
West Midlands	2454	4345	2895	*67*	9489	7282	*76*
Yorkshire & Humberside	2246	2162	1639	*76*	13513	12315	*91*
Wales	864	2874	1028	*36*	na	na	*na*

**Total**	**57340**	**38027**	**25918**	***68***	**78058**	**67712**	***87***

*reports were excluded from the matching exercise if patient information was missing or there were other anomalies.

na=not available

The matching exercise identified 199 individuals diagnosed with both hepatitis C and HIV ("co-diagnosed") of whom 47 were men who reported sex with another man (MSM) (table 2). Of the 47 co-diagnosed MSM, 16 were recorded as having injected drugs, having received a blood transfusion or blood factor products. These 16 MSM were therefore excluded from further analysis as they may not have acquired hepatitis C sexually.

**Table 2:** Probable route of HIV infection and hepatitis C risk for individuals identified as 'co-diagnosed' through matching

**Probable route of HIV infection**	**Probable route of hepatitis C infection**	**Number of "co-diagnosed"**
**Sex between men**	Injecting drug use	4
	Blood transfusion/product	1
	**No risk reported**	**31**
Sex between men & injecting drug use	Injecting drug use	2
	No risk reported	9
Injecting drug use	Injecting drug use	33
	Heterosexual intercourse	1
	No risk reported	61
Heterosexual intercourse	Injecting drug use	2
	Heterosexual intercourse	1
	No risk reported	26
Blood transfusion/product	Blood transfusion/product	5
	No risk reported	18
Not reported	Injecting drug use	1
	No risk reported	4

**Total**		**199**

**Figure 1:**
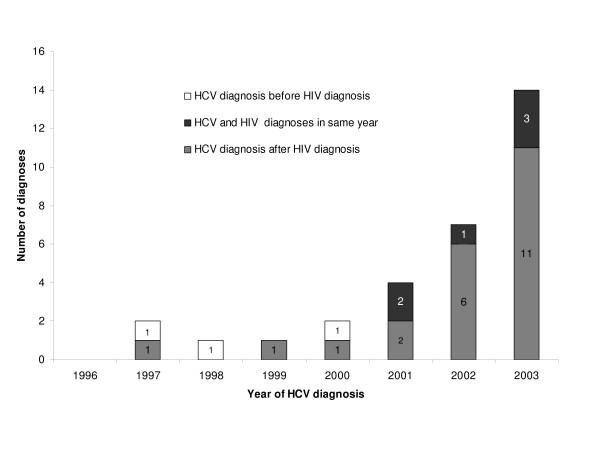
Number of MSM co-diagnosed with hepatitis C and HIV in England and Wales identified through the matching exercise, by year of hepatitis C diagnosis.

For the 31 remaining MSM with no other reported risk, median time between HIV and hepatitis C diagnoses was 26 months (IQR: 4–90 months); 22 were diagnosed with hepatitis C after their HIV diagnosis, three before and six in the same year. Median age at HIV diagnosis was 32 years and at hepatitis C diagnosis, 36 years. A rise in the number of co-diagnosed MSM was observed over time, from zero in 1996 to 14 in 2003 (figure 1).

**Figure 2:**
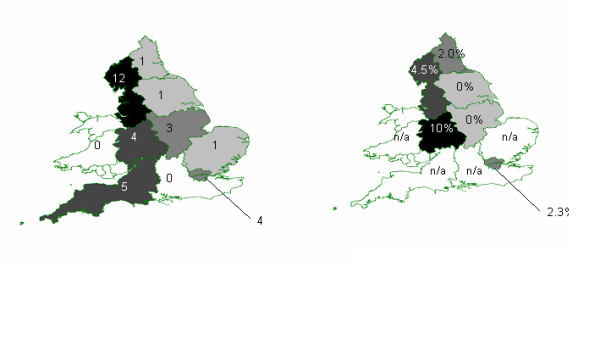
(a) Number of co-diagnosed MSM (1996–2003) and (b) proportion of diagnosed HIV-infected MSM with a positive hepatitis C test (2002–2003) by region of hepatitis C diagnosis/test in England and Wales, as identified through the matching exercise.

Twelve of the 31 MSM were diagnosed with hepatitis C in the North West, five in the South West, four in London, four in the West Midlands, three in East Midlands and three elsewhere (figure 2a). Where ethnicity was reported (n = 25), the majority (n = 23) were white, while two were black Caribbean. Where probable country of HIV infection was reported (n = 16), 75% (12) were infected with HIV in the UK

### Matching individual HIV diagnoses to reports from sentinel hepatitis C testing

Of the 78,058 individuals tested for hepatitis C in 2002 or 2003 by laboratories participating in the sentinel surveillance of hepatitis C testing, 87% (67,712/78,058) were eligible for inclusion in the matching exercise. Overall, 6% of the 78,058 individuals were tested at GUM clinics but records from GUM clinics represented 40% of the 10,346 excluded records (i.e. 82% of records from GUM clinics were excluded). This is because GUM clinics do not usually report soundex code/surname on laboratory test request forms. The number and proportion of hepatitis test results eligible for inclusion varied by region (table 1), reflecting both the sentinel nature of the surveillance system and the quality of patient information recorded.

The matching exercise identified 782 individuals who tested for hepatitis C in 2002–2003, who had also been diagnosed with HIV. Of these 782 individuals, 319 were MSM. Five of these men also had a history of injecting drug use and so were excluded from further analysis. Of the remaining 314 MSM with an HIV diagnosis who had had a hepatitis C test, 13 (4.1%) tested hepatitis C positive (95% confidence interval: 2.2%, 7.0%). Data for 2002 only: 2.8% (2/72) hepatitis C positive (95%CI: 0.3% to 9.7%). Data for 2003 only: 4.5% (11/242) hepatitis C positive (95%CI: 2.3% to 8.0%).

Median age at hepatitis C testing was 40 years and for HIV diagnosis, 33 years, which did not vary by hepatitis C test result. The percentage of MSM with diagnosed HIV who tested positive for hepatitis C varied between regions (figure 2b). Where ethnicity was reported on HIV reports, 94% (204/216) were white, again not varying by test result. Where probable country of HIV infection was reported, most MSM (134/155) were infected with HIV in the UK.

In 2003, 15,121 MSM with an HIV diagnosis were seen for treatment and care in E&W [16]. If 4.5% of these men tested positive for hepatitis C (assuming the same percentage tested positive in the overall MSM population seen for HIV-related care as seen here), we estimate that 680 MSM living with diagnosed HIV in E&W tested positive for sexually transmitted hepatitis C in 2003 (95%CI: 346 to 1208).

## Discussion

By matching individual hepatitis C and HIV diagnoses in England and Wales between 1996–2003, we identified 31 HIV-infected MSM with sexually transmitted hepatitis C infection, of whom only four were in London. Clinical studies suggest that this is a substantial underestimate [1,8]. For example, at one central London GUM clinic alone, 26 HIV positive MSM were diagnosed with sexually transmitted hepatitis C between 1997 and 2002. Across six large GUM clinics in London and Brighton, 225 HIV positive MSM with sexually transmitted hepatitis C were identified between October 2002 and August 2005 [1,8]. On the other hand, matching HIV diagnoses and hepatitis C tests from sentinel sites suggested that, in 2003, nearly five percent of MSM diagnosed with HIV who were tested for hepatitis C were found to be positive. Sexual transmission of hepatitis C was the likely route of infection. While this matching exercise was more successful, the true percentage may be underestimated since those attending GUM clinics, and likely to be at higher risk because of their sexual behaviours, were less likely to be included in the analysis because of a lack of patient identifiable information.

The percentage of HIV-infected individuals (mainly MSM) testing hepatitis C positive rose over time in a London GUM clinic, from 0.6% in 1996 to 9.3% in 2002 [1]. The estimates from our sentinel sites for 2002 and 2003 were more conservative (2.8% and 4.5%, respectively). Assuming that these percentages can be applied to all HIV positive MSM receiving treatment and care, we estimate that, in 2003, at least 680 MSM (95%CI: 346 to 1208) with diagnosed HIV tested positive for sexually transmitted hepatitis C in E&W.

Our analysis also shows that sexually transmitted hepatitis C among HIV-infected MSM is not confined to London and Brighton. Outside London, the number of HIV infected MSM with sexually transmitted hepatitis C infection was highest in the North West, which includes Manchester with a large MSM population and good reporting of both HIV and hepatitis C diagnoses (table 1) **[unpublished, HPA]**. Again however, these figures are likely to be underestimates due to a lack of reported patient identifiable information.

### Limitations of current surveillance systems

Our analyses are dependent on MSM being diagnosed with HIV and hepatitis C infection: both infections may be asymptomatic for a significant length of time and some may have died without being diagnosed with HIV and/or hepatitis C. The analyses also rely on MSM diagnosed with hepatitis C and/or HIV being reported to national surveillance systems. The hepatitis C laboratory data appeared to be poorly reported in London (table 1), relative to the number of diagnoses in other regions. Incomplete reporting within a region will affect the number of hepatitis C reports that can be matched to HIV diagnosis reports, and subsequently the number of co-diagnoses detected in that area. In addition, for the sentinel surveillance of hepatitis C testing, only two small London laboratories were included. These laboratories do not serve GUM clinics with large MSM populations and are not necessarily representative of all MSM testing for hepatitis C in London. On the other hand, many of the sentinel sites outside London were large laboratories in major provincial cities (Manchester, Leeds, Birmingham, Nottingham and Newcastle) so it is likely that a substantial number of MSM receiving HIV care outside London were included.

There are limitations to the matching process: individuals may be incorrectly matched or individuals may not be matched if information has been incorrectly recorded. Reports must also contain sufficient information for the matching process; overall nearly a third of the hepatitis C laboratory reports and more than ten percent of the hepatitis C test requests from sentinel surveillance could not be matched to HIV diagnoses because of missing patient information. This varied by region, and for hepatitis C diagnoses, over time. In the sentinel surveillance study, the majority of records for GUM clinic attendees, who may be at higher risk of acquiring HIV and sexually transmitted hepatitis C infection, had to be excluded because of a lack of reported soundex code/surname. Incomplete reporting will lead to underestimation and introduce bias, particularly in London. On the other hand, increases in hepatitis C testing and improvements in the reporting of patient information on laboratory diagnoses of hepatitis C may have led to improved ascertainment of co-diagnosed individuals over time. There may also be a bias in that hepatitis C testing might have been prompted by abnormal liver function tests or by injecting drug use that had not been disclosed at the time of HIV diagnosis.

## Conclusion

It was not possible to use current national surveillance systems to accurately monitor sexually transmitted hepatitis C infection among HIV-infected MSM across E&W. The number of HIV infected MSM with sexually transmitted hepatitis C infection was underestimated due to the limitations of the surveillance systems, particularly reporting of hepatitis C diagnoses, the matching process, and the sentinel nature of the hepatitis C testing data. Improved or enhanced surveillance methods (such as for lymphogranuloma venereum (LGV)) are needed to monitor sexually transmitted hepatitis C infection among HIV-infected MSM nationally, as well as in London. The recording of soundex codes and dates of birth on test request forms from GUM clinics would also improve the quality of surveillance data. Nonetheless, our study provides some insight into an area of gay men's sexual health where there is currently a paucity of information at a national level. Until now, studies have focused on MSM in London, but our results suggest that sexual transmission of hepatitis C infection has been reported among HIV positive MSM throughout E&W. This merits further investigation. An evaluation of the most appropriate hepatitis C testing algorithms for MSM with HIV also needs to be undertaken.

## Competing interests

The author(s) declare that they have no competing interests.

## Authors' contributions

SD collated the HIV diagnoses data, performed the matching exercises and drafted the manuscript. MB and LB provided the hepatitis C diagnoses and hepatitis C testing data respectively, and guided on their use in the matching exercises and interpretation of results. JE, KS, BE and MR all provided guidance on the study design, the interpretation of the resulting HIV and hepatitis C data, study limitations and conclusions, and drafting of the manuscript. All authors read and commented on manuscripts.

## Pre-publication history

The pre-publication history for this paper can be accessed here:


